# Phage Therapy in a 16-Year-Old Boy with Netherton Syndrome

**DOI:** 10.3389/fmed.2017.00094

**Published:** 2017-07-03

**Authors:** Pikria Zhvania, Naomi Sulinger Hoyle, Lia Nadareishvili, Dea Nizharadze, Mzia Kutateladze

**Affiliations:** ^1^Eliava Phage Therapy Center, Tbilisi, Georgia; ^2^G. Eliava Institute of Bacteriophages, Microbiology and Virology, Tbilisi, Georgia

**Keywords:** phage therapy, Netherton syndrome, staphylococcal infections, bacteriophages, pediatrics, antibiotic resistance, Eliava

## Abstract

Netherton syndrome (NS) is a rare autosomal recessive disorder, characterized by a classical triad of clinical features, including congenital ichthyosiform erythroderma, trichorrhexis invaginata, and atopic diathesis coupled with frequent bacterial infections (1). The genetic basis for the disease has been recently identified with mutations in gene SPINK5, which is involved in the regulation of formation of skin barriers. We report on a 16-year-old male with all the typical manifestations of NS, including atopic diathesis and ongoing serious staphylococcal infections and allergy to multiple antibiotics whose family sought help at the Eliava Phage Therapy Center when all other treatment options were failing. Treatment with several antistaphylococcal bacteriophage preparations led to significant improvement within 7 days and very substantial changes in his symptoms and quality of life after treatment for 6 months, including return visits to the Eliava Phage Therapy Center after 3 and 6 months of ongoing use of phage at home.

## Introduction

Netherton syndrome (NS) is a congenital erythroderma associated with a multitude of clinical abnormalities, first described by E. W. Netherton in 1958 in a girl with erythematous scaly dermatitis and “bamboo-like nodes” in her sparse fragile hair (2). This rare disease has been recently shown to involve mutations in gene SPINK5, which encodes a lymphoepithelial Kazal-type-related inhibitor, a serine protease inhibitor that is involved in the regulation of formation of skin barriers (3) It is particularly characterized by the triad of ichthyosis linearis circumflexa, a characteristic serpiginous migratory polycyclic eruption with double-edged scale; trichorrhexis invaginata, intussusception of the distal hair shaft into the proximal portion (“ball and socket” hair shaft deformity); and atopic diathesis, which can include asthma, atopic dermatitis, allergic rhinitis, anaphylactic reactions to foods (especially nuts, eggs, and fish), elevated immunoglobulin E, and/or hypereosinophilia. Failure to thrive, a mild delay in growth and development and short stature are also common, probably secondary to the large energy requirement related to the rapid skin turnover, increased insensible water and heat loss through the skin, and/or enteropathy with villous atrophy and malnutrition. Neurological deficits including seizure disorders and spastic diplegia have also been reported (4). Intellectual deficiency may occur.

Netherton syndrome patients are also at increased risk for immunological deficiencies. Impaired cellular and humoral immunity, with selective antibody deficiencies (hypo- or hyper-gammaglobulinemia) to bacterial polysaccharide antigens, may be present, resulting in recurrent sinopulmonary, skin, and systemic infections (5). There is no known cure for Netherton syndrome, so lifelong treatment is required. The disease is punctuated by periodic exacerbations. Skin colonization and secondary infections are common. Maintenance therapy typically consists of use of topical steroids and treatment of secondary infections with topical or systems antibiotics as appropriate.

### Bacteriophage Therapy

Bacteriophage therapy is an alternative therapy that has long been successfully used to treat *Staphylococcal* infections, in some parts of the world, including Georgia, and is drawing new interest worldwide in this era of antibiotic resistance (6). Bacteriophages are bacterial viruses that are highly specific for their host. The phage injects its DNA into the bacterial cell and takes over the host to produce phage progeny, soon lysing the cell to release phage which will go on to further infect new bacterial hosts. Phage therapy has been used been successfully used as antibacterial treatment in the former Soviet Union, France, and the USA, particularly in the pre-antibiotic era. Their use in humans is not considered to be experimental because of their extensive history of use there. Phage therapy is now drawing new interest worldwide in this era of antibiotic resistance (7). However, despite its rich history and long record of apparent safety, double blind controlled trials are only now beginning to be carried out to prove its efficacy up to modern standards for the treatment of various diseases. Compassionate use case studies are still an important source of information about its potential. The Eliava Phage Therapy Center was established 7 years ago by the Eliava Foundation, a non-profit organization made up of Eliava scientists and the Eliava Institute, to facilitate more direct focus on the use of phage, documentation of the consequences, and access to phage therapy for people coming from other countries and often continuing treatment once they return home.

## Presentation of the Case

MK, a 16-year-old French male with Netherton syndrome (NS), antibiotic resistant chronic *Staphylococcus aureus* skin infection and allergy to multiple groups of antibiotics, presented at the Eliava Phage Therapy Center in July of 2016.

The patient was born at full term by vaginal delivery with normal weight and length. A few days after birth, he developed skin erythema and scaliness, which became profound at the age of 2 months when he developed erythroderma and dehydration. A dry thin yellow film formed on his skin from the age of 2 months, slowly covering his whole body, and areas of scaly and peeling skin slowly increased. There were also blepharitis, changes to nails and hair, and keratosis of the palms and soles of the feet.

His medical history includes bronchial asthma, recurrent sinus and pulmonary infections, and periodic Staphylococcal skin infections, which lead to hospitalization and antibiotic therapy in a Children’s hospital in France. He has developed allergies against most groups of antibiotics (β-lactams, cephalosporins, and macrolids), ointment bases (vaseline and lanolin), and dexamethasone, in addition to food allergies (Table 1). His family notes that he is small in body-build and that his hair is sparse, short, and slowly growing. He has a PEG feeding tube to supplement feeding due to prominent dysphagia. The patient’s maternal aunt and grandmother local ichthyosis, and mother and father are healthy.

**Table 1:** List of medications and the type of allergic reaction.

Medication	Type of reaction
**Antibiotics**	
Cephalosporins (Cefuroxime)	Lyell’s syndrome
Penicillin	Puritis, erythema, hypertension
Macrolides	Puritis, erythema, urticaria
Sulfonamides	Erythema, urticaria
Aminoglycosides	Angioedema
**Non-steroidal anti-inflammatory drugs (Ibuprofen)**	Erythema, urticaria
Corticosteroids	Irritability, edema, erythema
**Ointments**	
Beeswax	Edema
Vaseline	Anaphylaxis
Lanolin	Erythema
Glycerin	Erythema
Paraffin	Erythema

### Clinical Examination

On examination, physical delays were noted: height 150 cm, weight—40 kg (both falling below the fifth percentile), while intellectual development was observed to be normal. His hair was lusterless, sparse, beaded, and easily broken, 4–5 cm in length, with sparse eyebrows and eye lashes. Skin changes noted were typical for NS: cracked, dry, skin, and the entire body covered with a serpiginous yellowish thick film, which was more prominent on the face and knees. The tightness of the skin restricted mobility of the knees and arms, preventing complete extension. Hyperkeratization was observed on both the palms and soles of the feet. He also had severe pruritus.

The complete blood count was normal, but ESR was slightly increased. Biochemical markers were normal. Bacteriological analysis of swabs taken from affected areas on the skin showed strong growth of *S. aureus*. This bacterial infection had been present for the last 4 years and was the main complication of the disease. The treatment of this infection was hindered due to antibiotic resistance of the strain and the patient’s individual allergies to multiple antibiotics. These were the main motivating factors for the patient’s family to seek alternative treatment and to approach our clinic in regards to phage therapy. We chose two *Staphylococcus*-targeting phage preparations, based on phage sensitivity testing—*Staphylococcus* bacteriophage, containing fully sequenced Sb1 phage (8), and Pyobacteriophage targeting *Staphylococcus* spp., *Streptococcus* spp., *E. coli, Pseudomonas aeruginosa*, and *Proteus* spp., both to be used *per os* and externally.

**Figure 1:**
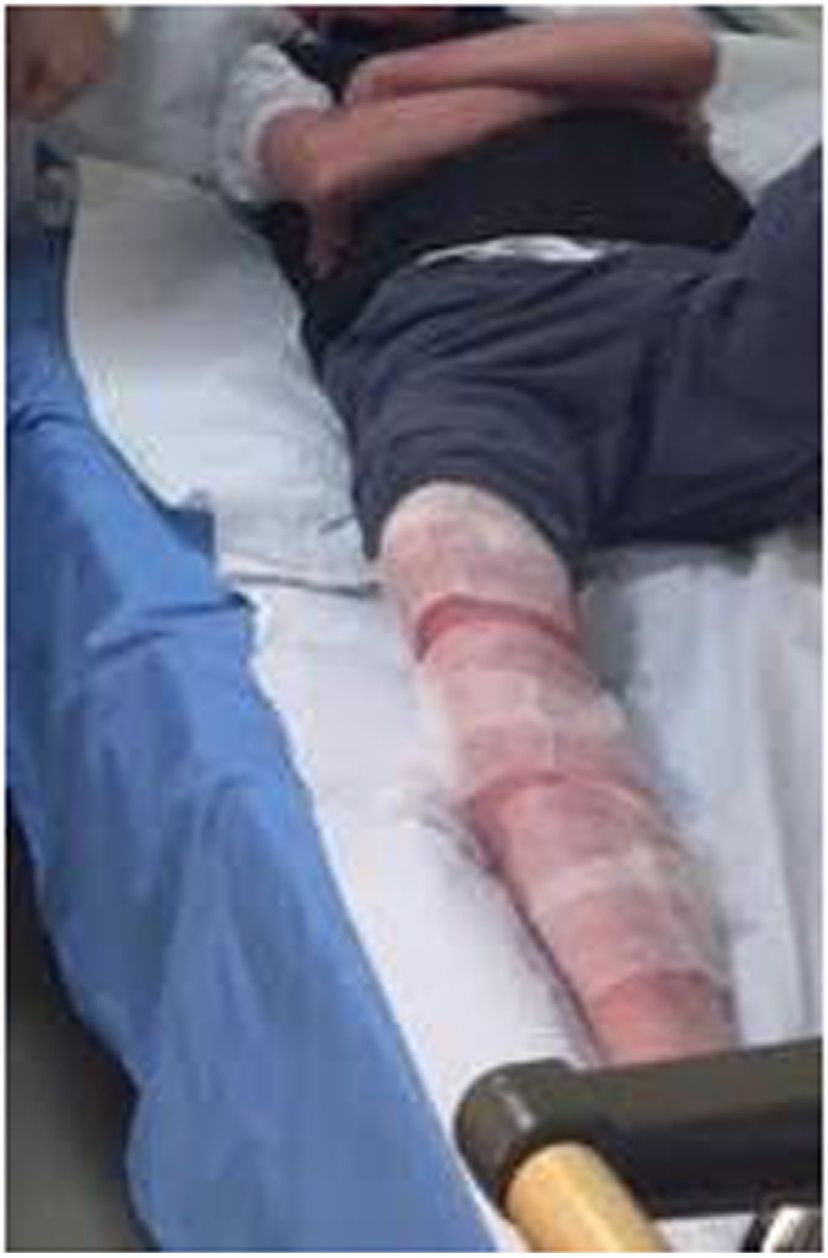
Local application of phage using gauze and liquid phage preparations.

Due to the patients’ allergic predisposition, phage was first only applied in a 1 cm^2^ area and was observed for 2 h. No adverse reactions were seen, and daily procedures were then carried out. First, the patient’s limbs were wrapped with sterile gauze (Figure 1) and liquid Pyobacteriophage was sprayed on with a sterile syringe to soak the gauze. After 20 min, the gauze was removed and a thin layer of *Staphylococcus* bacteriophage cream was applied. This cream was specially prepared in the Eliava Authorized Compounding Pharmacy using a hypoallergenic cream base, which the patient provided as previous use of this cream alone had not given him an allergic reaction. The cream was allowed to adsorb. The patient bathed with his regular hygenic regime each morning before the next treatment. Oral treatments with two phage preparations—Pyobacteriophage 10 ml once daily and *Staphylococcus* bacteriophage 10 ml once daily—were also taken after first neutralizing the stomach acid with 100 ml of “Borjomi” alkaline mineral water.

The initial phage treatment was carried out for 20 days, after which the patient took a 2-week pause and continued treatment for another 20 days. Repeat bacteriological testing performed after 3 months, identified *S. aureus* and *Streptococcus*, which was sensitive to “Fersis” (which targets *Staphylococcus* spp. and *Streptococcus* spp.) bacteriophage (Table 2). During the second 3 month period, the patient took phage for 2 week periods, with 2-week breaks in between treatment courses.

**Table 2:** Bacteriophage preparations (produced by Eliava Biopreparations Ltd.) used for treatment.

Phage preparation	Composition	Form	Titer	Local application	Oral	Eye	Nose
Pyo bacteriophage	Phage lysates of *Streptococcus* spp., *Staphylococcus* spp., *E. Coli, Pseudomonas aeruginosa*, and *Proteus* spp	Liquid	≈10^7^	+	+	+	+
*Staphylococcus* bacteriophage	Phage lysates of *Staphylococcus* spp.	Liquid, cream	≈10^7^	+	+	−	−
Fersis bacteriophage	Phage lysates of *Staphylococcus* spp., *Streptococcus* spp.	Liquid	≈10^7^	+	+	+	+

## Results

Visually, by the 7th day of treatment the infiltrated, hyperemic areas became smaller, the thickness of the yellowish film layer reduced, and mobility improved in the joints and areas of normal skin began to appear. No allergic reactions toward the phage preparations were seen.

The results of treatment were assessed in terms of the severity of NS symptoms, microbiological testing, and general quality of life:

- Netherton Area and severity assessment (NASA; each visit)
- Investigator’s Global Evaluation of Disease (IGED; each visit)
- *S. aureus* colonization of skin.

### Baseline Scores for NASA

Baseline scores for NASA were determined initially and compared with values obtained during treatment. Peak reductions from NASA baselines ranged from 18.4 to 11.2 after 3 months and 7.8 after 6 months of phage therapy. During the 6-month observation period, no eosinophilia was noted at baseline. No clinically significant abnormalities were observed in complete blood cell counts, hepatic function testing, electrolytes, or glucose monitoring.

### Microbiological Testing

Microbiological testing was done before intervention; qualitative bacterial cultures and culture sensitivities of the eyes, nostrils, and the worst, overtly infected lesions on the skin were obtained. At 3 and 6 months after the initiation of phage therapy, repeat swabs were obtained. The results showed a significant decrease in *S. aureus* in the eye and nostrils, while strong growth from the skin swab remained, indicating the need for prolonged treatment with periodic microbiological testing. Phage sensitivity profiles showed resistance to Pyobacteriophage after 3 months of treatment, after which an alternate phage cocktail was substituted to the treatment scheme.

Similar improvements were seen using the *IGED*, which was graded on a 5-point, 0–4 scale. A peak reduction of IGED scores of 55% was evident after 3 months treatment to 75% after 6 months treatment with phage.

On a follow-up visit 3 months later, regeneration of the skin had continued and the symptoms have not returned. Significant improvement was seen on the legs, especially around the knee joints, which were extremely tight and immobile, as well as the arms and face.

At a 6-month follow-up, the patients’ condition was stable. His skin continued to improve visually and he had not required hospitalization for generalized infection since starting phage therapy (See Figures S1 and S2 in Supplementary Material).

## Discussion

This patient had been hospitalized every 2 months due to infectious complications, and possible treatment had been limited because of bacterial resistance and drug allergies. General skin care was also severely limited because most cream bases also caused allergic reactions.

This patient’s case is a clear example of how bacteriophage therapy can be an effective treatment for controlling chronic infection in a patient with a genetic disease that has a predisposition toward infection. This is the first documented case of phage therapy in NS. Phage was applied externally in both liquid and cream forms and as an oral treatment, initially using two preparations, Pyobacteriophage and *Staphylococcus* bacteriophage. Over the first course of phage therapy, the patient’s condition significantly improved, indicating that periodic treatments with phage will be a viable alternative for infection control. Reports about immune system response to phage after prolonged use are a potential concern, as this patient will require prolonged periodic treatment, although recent studies show that this may not actually present a significant problem with therapeutic dosing (9). Another potential challenge is the possibility of changes in bacterial phage sensitivity. After a 1-month treatment course of external and internal phage use, the sensitivity profile did not change. After 3 months’ treatment, resistance developed to one of the phage preparations used. If resistance is observed, it can usually be overcome by substituting another preparation, as was done here, or the development of individual phage preparations, made on a custom basis.

### Phage Therapy

A total of 100 years after the discovery of bacteriophage, their role in medicine is of recurrent interest but still not secure. Viruses that attack bacteria were first observed by Twort and d’Herelle in 1915 and 1917. During the 1920s and 1930s the young Georgian microbiologist, G. Eliava, headed the Institute of Microbiology in Tbilisi. Eventually founding an institute in Tbilisi which continued on to be the largest producer of bacteriophage preparations in the former Soviet Union preparing bacteriophages to treat a wide range of topical, internal, and respiratory bacterial pathogens. After the fall of the Soviet Union, the institute met a lot of challenges. After years of work and international collaboration, the institute has re-established itself as a leader in bacteriophage research and its various applications in human, as well as in animal, plant, and environment (10). The use and study of phage therapy continues in the Georgia, Russia, and Poland and is increasingly being explored in the Western world as one potential weapon against the devastating rise of antibiotic resistant bacteria (11, 12).

The pharmacokinetics and pharmacodynamics of bacteriophage are complex and still being explored. Animal experiments in the early 1940s showed that phage are widely distributed throughout the body and easily cross the blood brain barrier (13). A more recent study in a pediatric population showed that orally administered phage is excreted into urine, stool, and CSF during administration and is quickly eliminated from the body, undetectable 3–5 days after cessation of treatment (14). There is much evidence that phages are safe for use (7, 10–12). There is no recorded anaphylactic reaction to phage in the literature. They are self-replicating and self-limited. The specificity of each for a very narrow group of targeted bacteria reduces side effects compared to other antimicrobials, which negatively affect the microbiome.

Despite the long history of successful use, phage therapy has not yet managed to re-enter Western medicine as a viable available treatment option due to a lack of randomized controlled trials, quintessential in the age of evidence based medicine. Many authors note the importance of fast tracking phage therapy, even if only for compassionate use in this era of antibiotic resistance, while definitive randomized controlled trials of various applications are being developed, funded, and carried out (7, 15–18).

The advantages of phage therapy are clear: the ability to easily treat many antibiotic-resistant microbes, antibacterial treatment in cases of drug allergy, and high tolerability with little or no side effects. This case represents the effective use of phage therapy for the treatment of infectious complications of a rare genetic disease, which had extremely limited treatment options. For this French patient, phage therapy represents an essential alternative to control frequent infections and reduce the severity of NS, thus significantly improving his quality of life.

## Informed Consent

Bacteriophage preparations are a registered drug in Georgia. Treatment with phage is not considered to be experimental in Georgia. Despite this, our patients are informed that bacteriophage preparations are not registered drugs in Europe and the United States. The parents of our patient agree to have their son’s information and photographs published in scientific articles.

## Ethics Statement

Phage therapy is not an experimental therapy in Georgia. The phage preparations used are registered drugs in Georgia, and the treating physicians are licensed in Georgia.

## Author Contributions

PZ, is a lead physician on the case, provided the background research about the disease and history of phage therapy. NH is a physician and phage specialist, wrote and edited the article. LN, is a supporting physician and phage therapy expert, contributed to writing. DN, is a chief physician and phage therapy expert, contributed to writing. MK, is a director of the Eliava Institute, intellectual proprietor, and phage expert, edited the article.

## Conflict of Interest Statement

The medical services provided were paid by the patient. All other authors declare no conflict of interest.

## Supplementary Material

Figure S1 Clinical images of patient before and after phage therapy; A, Face day 1; B, Face on 90 days; and C, Face on 180 days.

Figure S2 Clinical images of patient 2 before and after phage therapy. Anterior lower leg on day 1; on day 90; and anterior lower legs day 180.
