# Supplementary figures and images for: Phage Therapy in a 16-Year-Old Boy with Netherton Syndrome
Source: Front Med (Lausanne). 2017 Jul 3;4:94. doi: 10.3389/fmed.2017.00094 (PMC5494523; doi:10.3389/fmed.2017.00094)

Leg Before

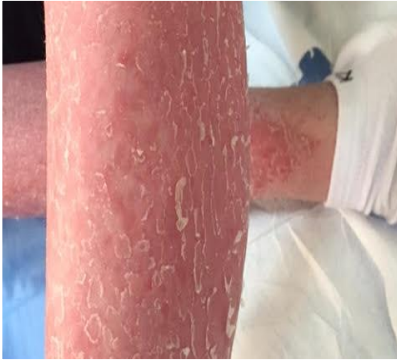

90 days of phage therapy

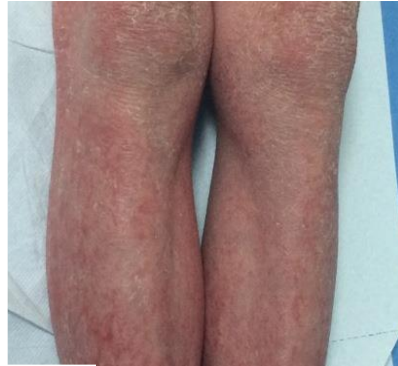

180 days of phage therapy

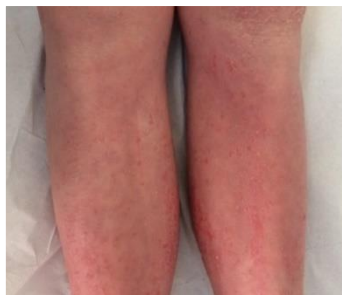

Supplement: Figure S1 — Clinical images of patient before and after phage therapy; A, Face day 1; B, Face on 90 days; and C, Face on 180 days. [file Image_1.PDF]
