# Supplementary material for: Phage Therapy in a 16-Year-Old Boy with Netherton Syndrome
Source: Front Med (Lausanne). 2017 Jul 3;4:94. doi: 10.3389/fmed.2017.00094 (PMC5494523; doi:10.3389/fmed.2017.00094)
Supplement: Figure S2 — Clinical images of patient 2 before and after phage therapy. Anterior lower leg on day 1; on day 90; and anterior lower legs day 180. [file Image_2.PDF]

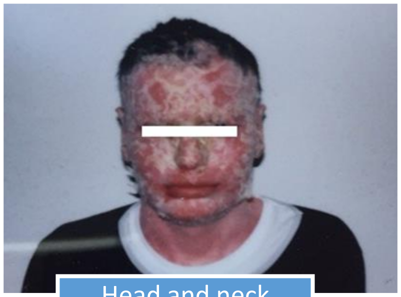

Head and neck before phage therapy

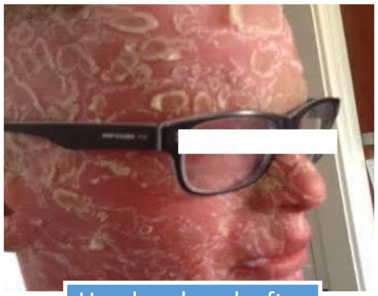

Head and neck after 90 days of phage therapy

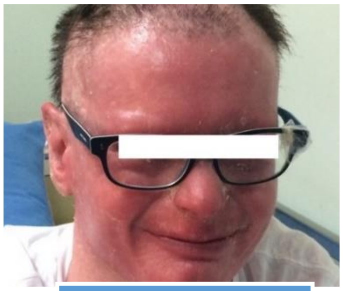

Head and neck after 180 days of phage therapy
